# TruSeq-Based Gene Expression Analysis of Formalin-Fixed Paraffin-Embedded (FFPE) Cutaneous T-Cell Lymphoma Samples: Subgroup Analysis Results and Elucidation of Biases from FFPE Sample Processing on the TruSeq Platform

**DOI:** 10.3389/fmed.2017.00153

**Published:** 2017-09-22

**Authors:** Philippe Lefrançois, Michael T. Tetzlaff, Linda Moreau, Andrew K. Watters, Elena Netchiporouk, Nathalie Provost, Martin Gilbert, Xiao Ni, Denis Sasseville, Madeleine Duvic, Ivan V. Litvinov

**Affiliations:** ^1^Division of Dermatology, McGill University Health Centre, Montreal, QC, Canada; ^2^Department of Pathology, Section of Dermatopathology, The University of Texas MD Anderson Cancer Center, Houston, TX, United States; ^3^Department of Pathology, McGill University Health Centre, Montreal, QC, Canada; ^4^Division of Dermatology, Université de Montréal, Montréal, QC, Canada; ^5^Division of Dermatology, Université Laval, Québec, QC, Canada; ^6^Department of Dermatology, The University of Texas MD Anderson Cancer Center, Houston, TX, United States; ^7^Division of Dermatology, Ottawa Hospital Research Institute, University of Ottawa, Ottawa, ON, Canada

**Keywords:** cutaneous T-cell lymphoma, mycosis fungoides, Sézary syndrome, prognostic markers, diagnostic markers, expression profiling, TruSeq

## Abstract

Cutaneous T-cell lymphomas (CTCLs) are a heterogeneous group of malignancies with courses ranging from indolent to potentially lethal. We recently studied in a 157 patient cohort gene expression profiles generated by the TruSeq targeted RNA gene expression sequencing. We observed that the sequencing library quality and depth from formalin-fixed paraffin-embedded (FFPE) skin samples were significantly lower when biopsies were obtained prior to 2009. We also observed that the fresh CTCL samples clustered together, even though they included stage I–IV disease. In this study, we compared TruSeq gene expression patterns in older (≤2008) vs. more recent (≥2009) FFPE samples to determine whether these clustering analyses and earlier described differentially expressed gene findings are robust when analyzed based on the year of biopsy. We also explored biases found in FFPE samples when subjected to the TruSeq analysis of gene expression. Our results showed that ≤2008 and ≥2009 samples clustered equally well to the full data set and, importantly, both analyses produced nearly identical trends and findings. Specifically, both analyses enriched nearly identical DEGs when comparing benign vs. (1) stage I–IV and (2) stage IV (alone) CTCL samples. Results obtained using either ≤2008 or ≥2009 samples were strongly correlated. Furthermore, by using subgroup analyses, we were able to identify additional novel differentially expressed genes (DEGs), which did not reach statistical significance in the prior full data set analysis. Those included CTCL-upregulated *BCL11A, SELL, IRF1, SMAD1, CASP1, BIRC5*, and *MAX* and CTCL-downregulated *MDM4, SERPINB3*, and *THBS4* genes. With respect to sample biases, no matter if we performed subgroup analyses or full data set analysis, fresh samples tightly clustered together. While principal component analysis revealed that fresh samples were spatially closer together, indicating some preprocessing batch effect, they remained in the proximity to other normal/benign and FFPE CTCL samples and were not clustering as outliers by themselves. Notably, this did not affect the determination of DEGs when analyzing ≥2009 samples (fresh and FFPE biopsies) vs. ≥2009 FFPE samples alone.

## Introduction

Cutaneous T-cell lymphomas (CTCLs) represent ~4–8% of all non-Hodgkin’s lymphomas and are characterized by infiltration of malignant T lymphocytes into the skin (1). Most patients first present with stage I disease, limited to the skin, which can either follow an indolent course (in 70–80% of cases) or progress to a potentially devastating, deadly malignancy with a median survival of <3 years (2). The diagnosis of CTCL is rather challenging for several reasons. First, mycosis fungoides (MF) and Sézary syndrome (SS), the most recognized variants of CTCL, can have variable presentation (3). Second, other common and rare benign inflammatory dermatoses can mimic CTCL and *vice versa*. Classically, MF may present with centrally distributed erythematous patches and plaques that are not specific to CTCL and are commonly misdiagnosed as chronic eczema, psoriasis, pityriasis rubra pilaris, drug eruptions, and dermatophyte infections. Finally, histopathological analysis of skin biopsies and PCR evaluation of T-cell receptor clonality lacks sensitivity in early MF patients and in erythrodermic disease. Unfortunately, current time to CTCL diagnosis from its initial presentation averages ~6 years (4).

Factors involved in the pathogenesis and prognostication of CTCL have emerged from recent epidemiological (5–8), karyotype/chromosomal (9–23), exome sequencings (24–28), gene and microRNA expression profiling studies (3, 29–41), but remain incomplete and poorly elucidated. The lymphocyte precursor population was proposed to be different between MF (skin resident memory T lymphocytes) vs. SS (skin tropic central memory T lymphocytes with wide tropism) (42–45). Importantly, significant disease heterogeneity was noted on a molecular level, and genetic alterations in MF/SS were often not replicated between different studies. Pathways that are believed to be involved in CTCL pathogenesis include T-cell function/signaling/differentiation, *JAK/STAT/NF-κB* signaling, cytokine production, chromatin remodeling, cell cycle checkpoint regulation, DNA repair, as well as cancer testis and embryonic stem cell signaling and function (24, 25, 28, 46). The goal of discovery and validation of prognostic biomarkers for disease progression and patient survival remains critical to help identify the minority of stage I MF patients, who will eventually progress to advanced disease (~20–30% of patients). Poor disease outcome may be heralded by high expression of *TOX, GTSF1, NOTCH1, CCR4, ITK, FYB, SYC1, LCK* or *miR155, miR21, and let-7i* microRNAs (26, 31, 39, 47).

Recently, we analyzed using Illumina’s TruSeq targeted RNA gene expression platform a new cohort of 157 patients, with biopsy-confirmed CTCL and compared it to a cohort of patients with normal skin and benign skin conditions (41). A number of patients in this study provided longitudinal biopsy samples (41). Analyzed samples included (A) 29 formalin-fixed paraffin-embedded (FFPE) tissues from benign inflammatory dermatoses and skin tag biopsies (1 sample per patient; 7 skin tag samples and 22 benign inflammatory dermatoses samples); (B) 134 FFPE samples of lesional CTCL skin from 110 patients; and (C) an additional 18 samples of freshly obtained and liquid nitrogen snap-frozen skin samples from a different group of CTCL patients. We processed 181 skin biopsy samples either freshly obtained or FFPE using TruSeq platform, capturing 284 genes that were previously identified as important for CTCL diagnosis and/or prognosis (32, 48). We identified 75 statistically significant differentially expressed genes (DEGs) between benign skin samples and either all CTCL or stage IV CTCL samples (41) and validated a number of our previous diagnostic and prognostic expression markers (3, 41).

However, we noticed non-trivial heterogeneity when performing clustering based on the TruSeq gene expression data, where early-stage CTCL samples and benign samples were admixed in the same clusters with the stage IV advanced CTCL disease. We hypothesized that this could be due to differences in TruSeq library sequencing depth and/or variation in the quality of the FFPE samples obtained during 2007–2008 (older) vs. 2009–2012 (more recent) years. Indeed, recent samples that were freshly obtained and snap frozen had comparable total number of sequencing reads (400–1,000 K reads), while older FFPE samples had often <300 K sequencing reads (41). In addition, we observed that freshly obtained snap-frozen CTCL samples were often tightly grouped in the same cluster, independent of their disease stage (41). This may indicate that TruSeq gene expression analysis may be affected by intrinsic biases based on the very natures of the samples analyzed (e.g., FFPE vs. fresh-frozen biopsies).

Notably, these variables (i.e., old vs. new; FFPE vs. freshly obtained snap frozen) were not formally evaluated in the prior publication but may contribute to the observed heterogeneity. These variations contribute toward a larger problem, known as the batch effect, in the field of gene expression-based analyses that utilize TruSeq, RNA-Seq, gene expression microarrays, and other approaches to identify DEGs. Differences in preprocessing, sequencing runs, technicians/centers, date of experiments, populations, and experimental design can account for heterogeneity that will remain despite normalization and use of control samples. Potential consequences of batch effect include reduction of statistical accuracy, introduction of spurious DEGs, and discrepancies between observed and true correlations (49). Several techniques can be used to minimize batch effects without removing true signals including surrogate variable analysis (50), ComBat (51), and principal component-based approaches (i.e., EIGENSTRAT among others) (52).

In this study, we aimed to characterize TruSeq gene expression patterns separately in older (≤2008) vs. more recent (≥2009) FFPE samples to determine whether clustering analyses results display robustness when compared to the full data set. We also explored sample processing biases (old vs. new and FFPE vs. freshly obtained snap frozen).

## Materials and Methods

### Patients and Samples

As described before (41), all patients were enrolled in the study in accordance with the IRB-approved protocols: PA12-0267, PA12-0497, and Lab97-256 at the MD Anderson Cancer Center (MDACC) and A09-M106-13A and 13-201-GEN at McGill University/McGill University Health Centre (MUHC). This study was carried out in accordance with the recommendations of the Research Ethics Board of the McGill University/MUHC with written informed consent from all subjects in accordance with the Declaration of Helsinki. This study was carried out in accordance with the recommendations of the MDACC Research Ethics Board, which exempted us from obtaining written informed consent from patients, who earlier signed a hospital consent allowing their stored biopsy samples to be used for research.

### Data Acquisition

Processed TruSeq data from Litvinov et al (41) were re-analyzed in this study based on transcripts per million (TPM) and RNA integrity number (RIN) parameters. Raw data were deposited in the NCBI SRA, accession number SRP114956. We separated CTCL FFPE samples obtained from the MDACC into two subgroups: older (≤2008) vs. more recent (≥2009).

### Clustering

Unsupervised hierarchal clustering was performed in R, using packages stats, cluster, and gplots. Pairwise dissimilarity (distance) matrix was calculated using Gower’s method, which performs well in the case of incomplete/missing data when compared to other methods (53). Clusters were obtained using Ward’s clustering method and criteria (54). Silhouette plots followed by visual inspection (to ensure appropriately sized clusters) were used to assess clusters and subclusters divisions. We repeated similar comparisons for all samples, benign samples vs. stage IV CTCL disease, and early (stage ≤IIA) vs. intermediate (stages IIB and III) vs. advanced (stage IV) CTCL.

### Principal Component Analysis (PCA)

Principal component analysis was performed on scaled, centered TPM data using package pcaMethods (55). Probabilistic PCA was used to account for missing data. Score plots of principal components 1 and 2 were generated.

### Statistical Analyses

Differences in mean TPMs were determined using two-tailed Ward’s *t*-test. Power analysis showed an 86% power to detect a twofold expression change at a significance level of 0.05 for the comparison between the smallest subgroups, with complete data points. Correlations were computed using Spearman’s rho, on log-2 ratios. Mean RINs were compared using a Bayesian analysis with Markov Chain Monte Carlo (MCMC) simulations, using R package rjags; at least 100,000 iterations were performed to estimate *p* values.

## Results

### Subgroup Clustering Analysis of All Samples

**Figure 1:**
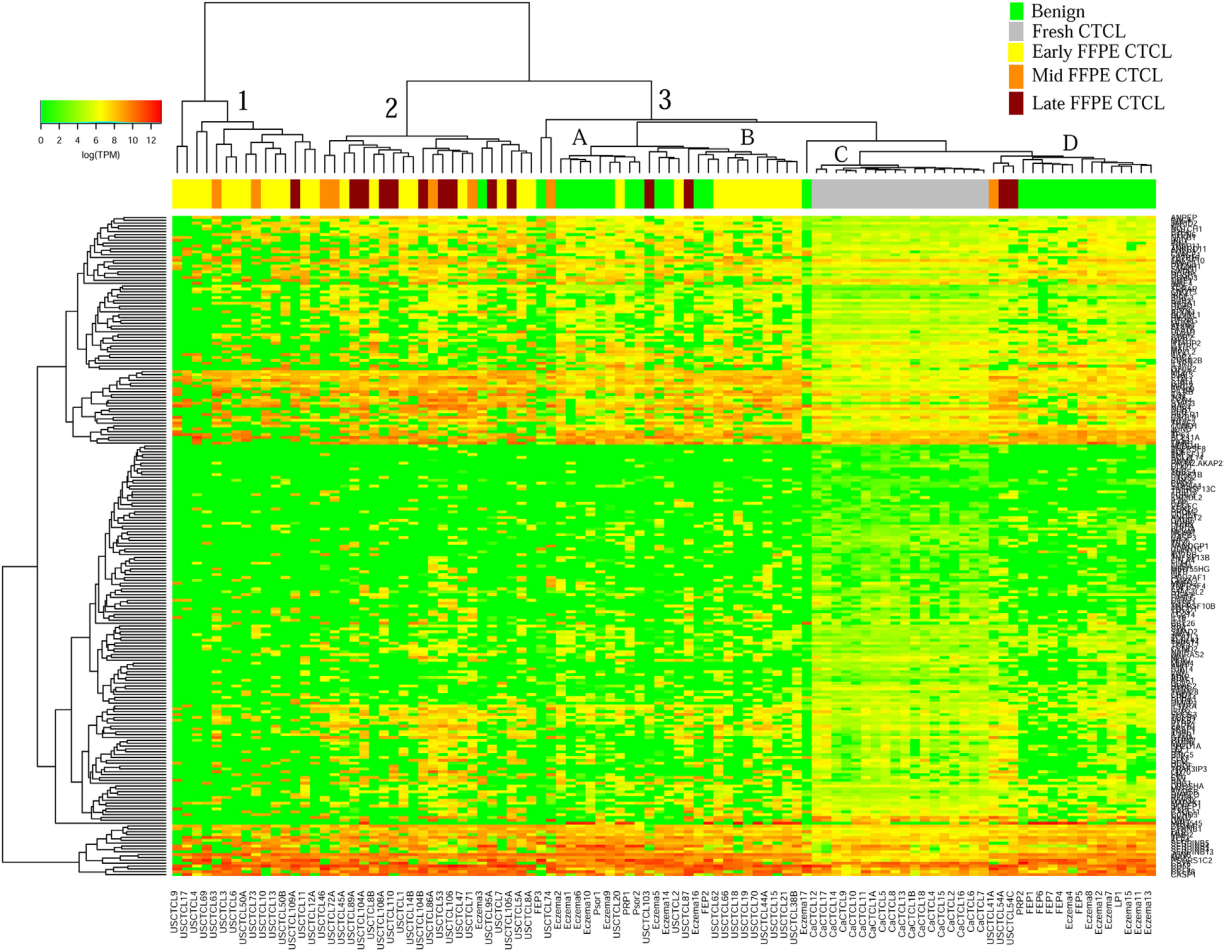
Unsupervised hierarchical clustering analysis based on TruSeq targeted RNA gene expression analysis of 284 select genes in benign inflammatory dermatoses (green), freshly obtained and snap-frozen cutaneous T-cell lymphoma (CTCL) samples (gray), and ≤2008 formalin-fixed paraffin-embedded (FFPE) CTCL samples (early stage, yellow; mid stage, orange; and late stage, dark red). A color key refers to gene expression in log(transcripts per million).

**Figure 2:**
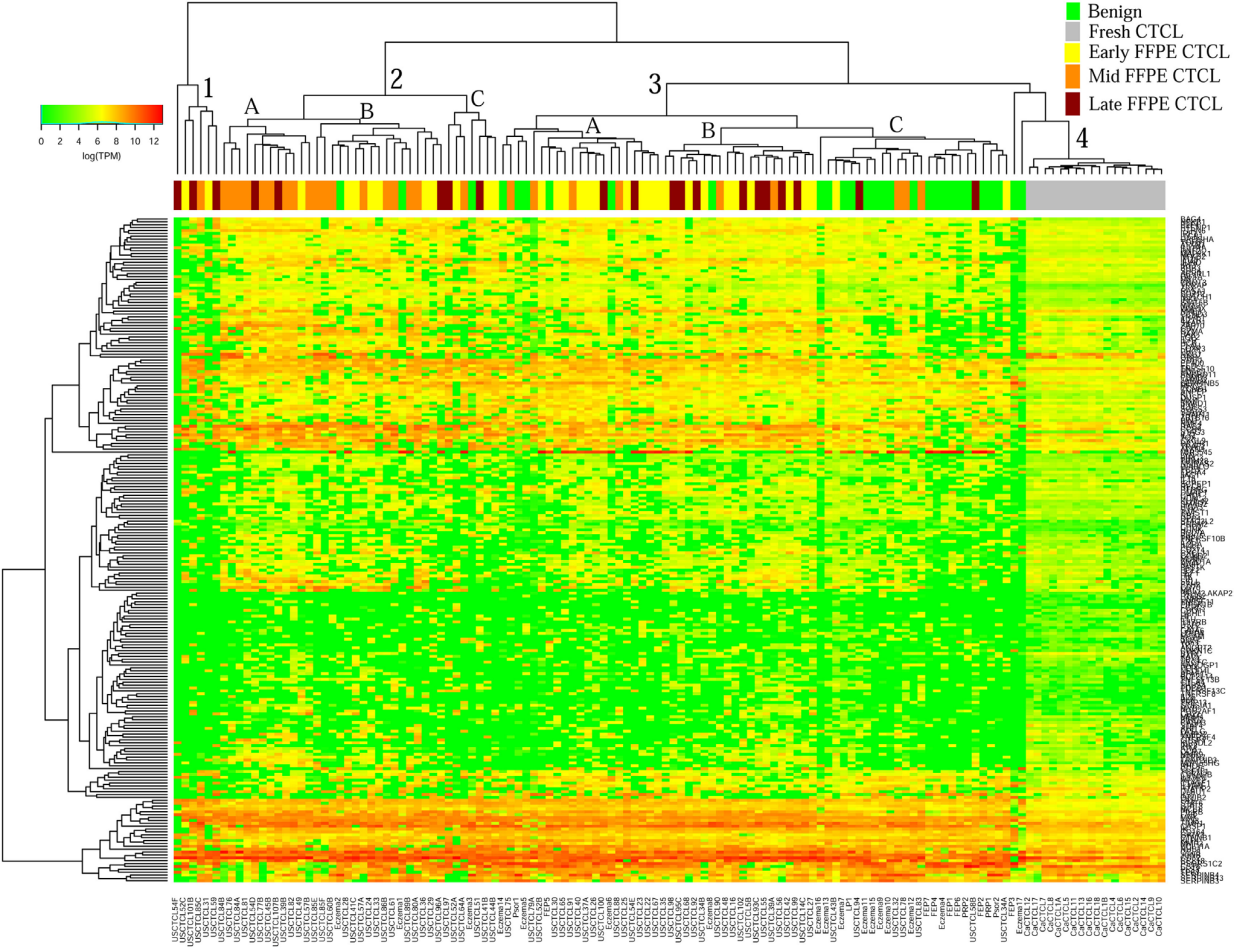
Unsupervised hierarchical clustering analysis based on TruSeq targeted RNA gene expression analysis of 284 select genes in benign inflammatory dermatoses (green), freshly obtained and snap-frozen cutaneous T-cell lymphoma (CTCL) samples (gray), and ≥2009 formalin-fixed paraffin-embedded (FFPE) CTCL samples (early stage, yellow; mid stage, orange; and late stage, dark red). A color key refers to gene expression in log(transcripts per million).

We previously noted that the ≤2008 FFPE samples had significantly decreased number of sequence reads per sample when compared to the ≥2009 samples (mean 103,406 ± 96,620 vs. 437,218 ± 550,840 reads, respectively). Therefore, we repeated unsupervised hierarchical clustering for benign samples (skin tags and benign inflammatory dermatoses), fresh liquid nitrogen snap-frozen CTCL samples, and either ≤2008 or ≥2009 FFPE CTCL samples. For ≤2008 FFPE sample analysis (Figure 1), we observed three major clusters. Cluster 1 comprised exclusively the FFPE CTCL samples, mostly early-stage (≤IIA) (12/33), along with two mid-stage (IIB and III) (2/8) and one late-stage (IV) (1/14) disease. In Cluster 2, 21 of 22 samples were from CTCL patients representing advanced stages (mid = 4/8 and late = 9/14), along with one eczema sample and a number of early-stage CTCL samples (8/33). Cluster 3 formed multiple subgroups (~4) that comprised mostly benign samples (28/29) and fresh CTCL samples, along with many early-stage and mid-late stage FFPE CTCL samples (early = 13/33, mid = 2/8, and late = 4/14). As previously discussed (41), one of the subgroups encompassed all fresh CTCL samples, which tightly clustered together (18/18). Two of the subgroups contained mostly benign samples, while the last one had early-stage FFPE CTCL samples. For ≥2009 FFPE samples (Figure 2), we noted two small clusters and two larger clusters. The first small cluster on the left panel (Cluster 1) contained six FFPE CTCL samples (two early, one mid, and three late). The second small cluster on the right (Cluster 4) included 18/18 fresh CTCL samples similarly to our previous analyses along with 2 benign biopsies. The first large cluster on the center left panel (Cluster 2) exhibited significant molecular disease heterogeneity. The first subgroup (A) had primarily mid-stage (*n* = 9) CTCL skin biopsies, one early and two late-stage samples, while the other two subgroups (B and C) were very heterogeneous with respect to their composition. For the second large cluster on the center right panel (Cluster 3), a similar admixture was observed with three subgroups, one subgroup being comprised primarily benign skin samples (C) and the other two containing predominantly early (A) and advanced (B) stage CTCL disease samples.

### Subgroup Clustering Analysis of Healthy Skin/Benign Inflammatory Dermatoses Samples vs. Stage IV CTCL Samples

**Figure 3:**
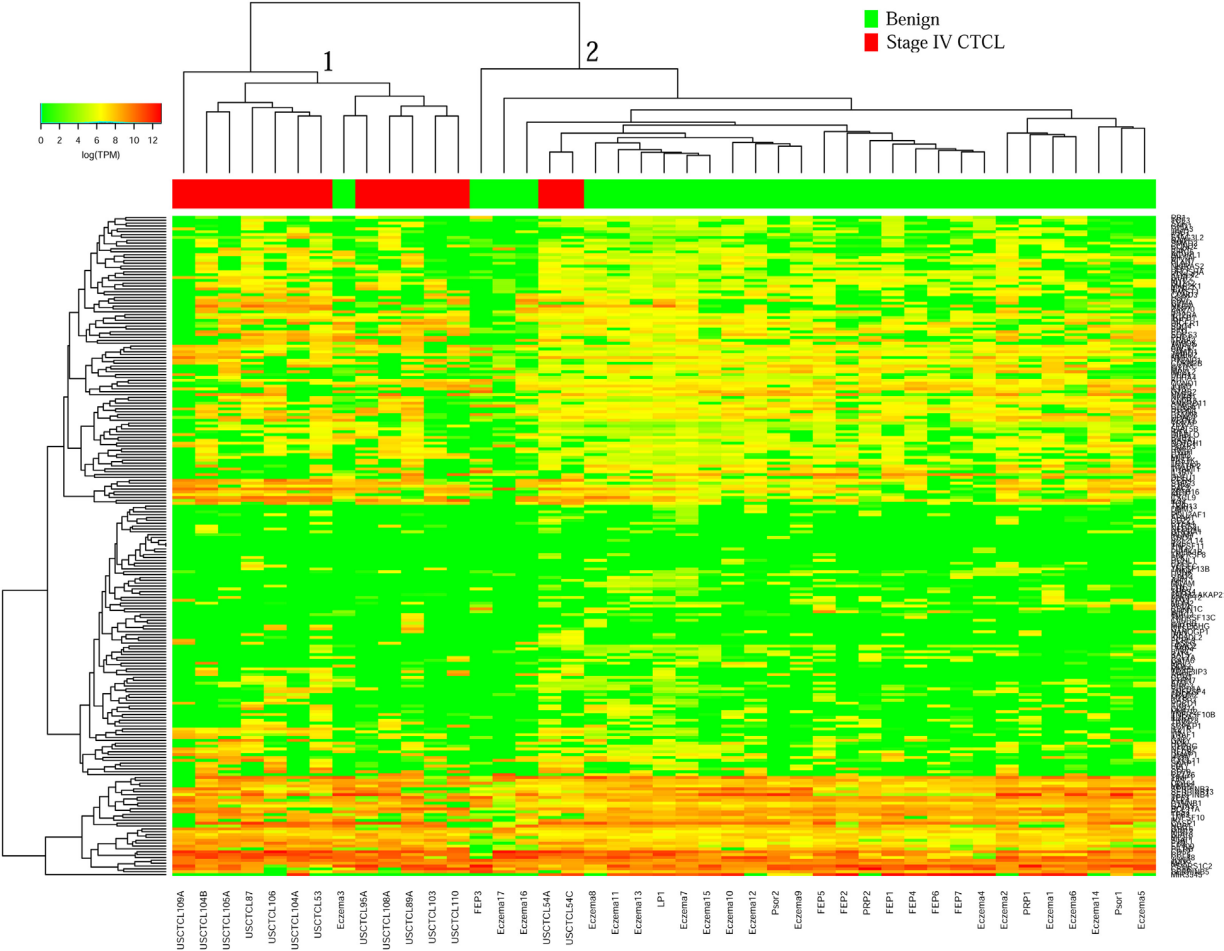
Unsupervised hierarchical clustering analysis based on TruSeq targeted RNA gene expression analysis of 284 select genes in benign inflammatory dermatoses (green) vs. ≤2008 stage IV formalin-fixed paraffin-embedded cutaneous T-cell lymphoma (CTCL) samples (red). A color key refers to gene expression in log(transcripts per million).

We then performed unsupervised hierarchical clustering for benign samples (which included skin tags and benign dermatoses that often clinically mimic CTCL) vs. stage IV CTCL disease. Similarly, two analyses were performed for ≤2008 and ≥2009 FFPE biopsies. In the case of ≤2008 samples (Figure 3), there were two major clusters that separated quite well these biopsies based on gene expression changes. Cluster 1 had 13 samples, 12 of which were stage IV CTCL disease (including 12/14 of total stage IV CTCL samples) and 1 sample form a patient with chronic eczema. Cluster 2 contained 30 samples in total and comprised mostly benign dermatoses and skin tags (*n* = 28) and 2 stage IV CTCL samples.

**Figure 4:**
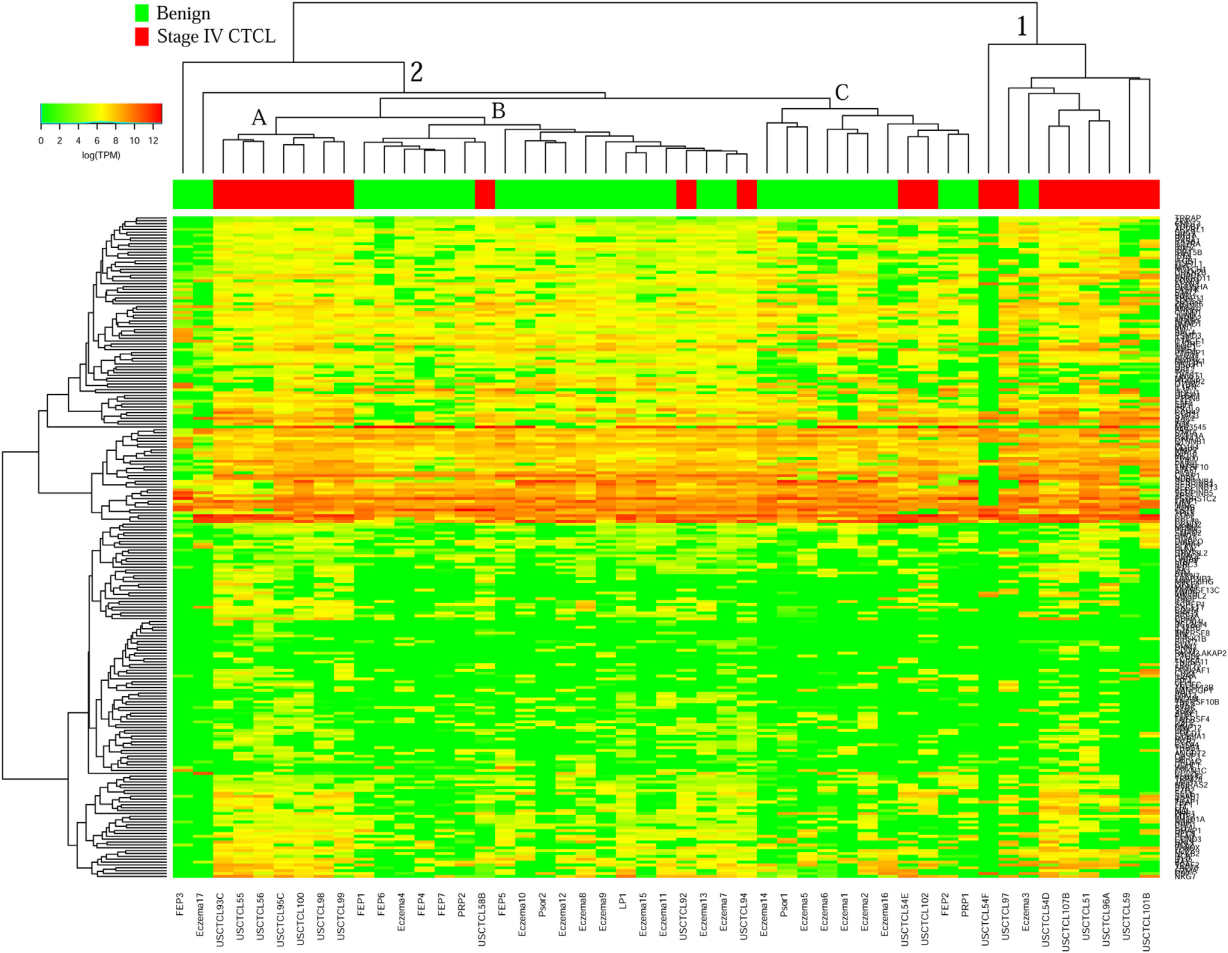
Unsupervised hierarchical clustering analysis based on TruSeq targeted RNA gene expression analysis of 284 select genes in benign inflammatory dermatoses (green), vs. ≥2009 stage IV formalin-fixed paraffin-embedded cutaneous T-cell lymphoma (CTCL) samples (red). A color key refers to gene expression in log(transcripts per million).

Surprisingly, for ≥2009 samples (Figure 4), greater overall heterogeneity was observed. However, we noted one small cluster in the right panel and one large cluster with three subgroups in the center. Cluster 1 (right panel) had nine samples, eight of which were stage IV samples (8/20 total stage IV CTCL samples). Cluster 2 was subdivided into three subgroups, where 2A samples (*n* = 7) with advanced CTCL disease tightly clustered together, while 2B (*n* = 20) and 2C (*n* = 11) samples included primarily benign dermatoses and skin tags (85 and 82%, respectively, for each subcluster).

### Identification of Differentially Expressed Genes (DEGs) in All Samples Using Subgroup Analyses Based on the Year of Biopsy and Benign vs. Malignant Nature of Samples

We then analyzed our full data set, by performing a Wald’s *t*-test to compare either benign dermatoses vs. (1) all CTCL samples or (2) stage IV CTCL. In our initial report (41), we identified important differentially expressed genes (DEGs) including *TOX, FYB, LEF1, CCR4, ITK, EED, POU2AF, IL-26, STAT5, BLK, GTSF1, PSORS1C2, CD70*, and *STAT* signaling genes; *LTA, NFKB1, NFKB2*, and *IL-15*; and other inflammatory cytokines. In this study, we repeated the analysis of the FFPE samples obtained ≤2008 vs. ≥2009.

As presented in Table 1, our analysis revealed 54 DEGs (*p* < 0.05), when ≤2008 stage I–IV CTCL or ≤2008 stage IV CTCL samples were compared to benign skin samples. This list included 47/75 DEGs that were enriched in the initially reported full data set (41). New highlighted CTCL-upregulated targets in this analysis included *BCL11A, SELL, IRF1, SMAD1, CASP1*, and *BIRC5*, while *THBS4* was upregulated in benign skin samples. For ≥2009 samples, 41 significant DEGs (*p* < 0.05) were found when freshly obtained and for ≥2009 samples, FFPE CTCL biopsies were analyzed together in a similar way (Table 2). Importantly, the same 41 DEGs were identified using only the ≥2009 FFPE samples alone (i.e., excluding the freshly obtained biopsies from this analysis). In the latter analysis, four additional CTCL-upregulated DEGs (*EP400, NFKB1, TRRAP*, and *MAX*) were revealed as being statistically significant (Table 2).

**Table 1:** Genes with statistically significant differences in expression both between benign skin dermatoses vs. all ≤2008 CTCL samples (left panel) and between benign skin lesions vs. ≤2008 stage IV CTCL samples (right panel).

Genes	Average of benign [transcripts per million (TPM)]	Average of all CTCL (TPM)	log2 ratio (all CTCL vs. benign)	*p* Value (all CTCL vs. benign)	Average of stage IV CTCL (TPM)	log2 ratio (stage IV CTCL vs. benign)	*p* Value (stage IV CTCL vs. benign)
*CCR7*	91.6375	1,571.482	4.100044	0.049964	748.8	3.03057	0.001363
*LTA*	189.1833	2,300.565	3.604131	0.006095	1,473.638	2.961525	0.015005
*CD70*	813.88	8,678.515	3.41456	0.001653	7,529.575	3.20968	0.003482
*ITK*	140.1125	1,447.92	3.369324	4.33E−05	1,136.309	3.019698	0.002318
*TOX*	410.12	3,459.284	3.076355	2.65E−08	4,502.264	3.456533	0.000852
*LEF1*	161.0417	1,283.983	2.995121	0.000612	1,056.588	2.713906	0.00929
*CCR4*	1,375.88	7,700.756	2.484645	6.15E−05	8,003.146	2.540213	0.004464
*IL21R*	256.3111	1,344.811	2.391435	0.000208	1,600.418	2.642481	0.004746
*FYB*	1,337.875	6,587.762	2.299845	5.97E−08	8,462.607	2.661159	0.002439
*TRAF1*	221.0818	1,055.033	2.254636	2.77E−05	1,357.833	2.618654	0.010125
*ZAP70*	915.675	4,096.65	2.161537	2.87E−05	4,007.87	2.129928	0.040324
*IL7R*	523.8217	2,339.003	2.158746	0.001063	2,376.782	2.181862	0.04109
*SELL*	1,160.508	5,064.55	2.125677	0.003904	5,525.386	2.251318	0.041623
*ZBTB16*	1,100.665	4,684.436	2.0895	0.000107	3,581.977	1.70238	0.012754
*CDKN2B*	476.1875	2,021.157	2.08558	0.000255	1,735.944	1.866119	0.019539
*NFKB2*	320.1294	1,356.579	2.083246	0.000131	1,336.467	2.061697	0.045613
*MMP9*	149.2333	609.0611	2.029017	0.00312	708.9625	2.24814	0.013971
*PILRB*	2562.259	10,430.87	2.025371	1.22E−05	8,270.423	1.690545	0.039467
*IL32*	2,781.636	11,217.26	2.011714	2.44E−05	10,043.51	1.852258	0.00489
*STAT1*	1,714.134	6,868.114	2.002434	2.52E−08	6,162.807	1.846107	0.000347
*STAG3*	1,118.487	4,312.054	1.946827	5.32E−07	4,394.586	1.974179	0.001446
*EED*	1,110.654	4,229.108	1.928944	6.99E−06	3,032.208	1.44896	0.039
*LCP2*	535.9733	2,010.365	1.907224	2.45E−06	2,396.473	2.160679	1.96E−05
*EZH2*	466.14	1,715.855	1.880092	6E−07	1,339.258	1.522599	0.004833
*NME4*	3075.109	11,318.73	1.880003	0.000737	9,229.838	1.585668	0.022129
*PTPN6*	746.3091	2,663.14	1.835283	8.96E−08	2,509.582	1.749602	0.005967
*IRF7*	841.7043	2,956.561	1.812534	0.008642	2,596.145	1.624986	0.01711
*IRF4*	192.3	652.3552	1.762299	0.000497	493.4556	1.359561	0.041528
*KLHL42*	136.4864	462.55	1.760853	0.004419	388.9889	1.510972	0.015901
*RASA1*	338.0591	1,118.067	1.725659	2.71E−06	951.1375	1.492378	0.014139
*CXCL9*	2,507.891	8,268.812	1.721205	0.00098	9,713.692	1.953545	0.010393
*MTF2*	217.3882	704.213	1.695738	0.000696	728.3556	1.744369	0.009782
*BIRC5*	338.0077	1,056.554	1.644238	0.007351	1,149.533	1.76592	0.006444
*RAC2*	4,323.963	13,309.44	1.622024	0.000209	10,852.03	1.327539	0.027541
*MAP2K1*	412.5632	1,209.649	1.551901	0.000416	1,143.84	1.471198	0.021317
*EP400*	1,879.341	5,316.209	1.500171	3.7E−05	4,616.664	1.296624	0.009013
*TRAF2*	2,425.25	6,748.69	1.476474	0.000693	5,677.97	1.227242	0.017582
*SUZ12*	340.216	943.5389	1.471631	1.3E−06	963.2273	1.501425	0.00096
*JARID2*	698.2037	1,913.181	1.454253	1.7E−05	1,853.242	1.408331	0.00949
*TGFB1*	394.75	1,065.353	1.43232	3.62E−05	1,000.375	1.34153	0.008951
*SOCS3*	1,008.825	2,703.836	1.422332	0.008306	3,391.209	1.749124	0.019409
*MCL1*	1,349.318	3,542.977	1.392732	7.2E−07	2,977.636	1.141937	0.005681
*MYC*	380.7762	996.8	1.388361	0.00144	1,117.25	1.552937	0.005836
*STAT5B*	210.0545	538.47	1.358102	0.002673	559.9091	1.414429	0.008889
*WWOX*	953.3938	2401.292	1.332667	0.002271	2,353.325	1.303557	0.048617
*BCL11A*	6,489.772	1,5869.53	1.29002	0.004204	15,268.44	1.234313	0.026839
*ANPEP*	820.7069	1,975.305	1.267136	0.000199	1,934.646	1.237131	0.019324
*SMAD1*	829.5667	1,947.6	1.231268	0.001198	2,076.155	1.323484	0.045054
*CCL5*	18,304.19	42,227.59	1.206012	0.006711	43,568.9	1.251125	0.006718
*IRF1*	690.6826	1,493.441	1.112546	0.005323	1,314.473	0.928389	0.032976
*NOTCH1*	393.3417	847.8263	1.107986	0.003446	834.7364	1.085538	0.046229
*CD52*	39,313.92	79,582.7	1.017415	0.001914	95,658.19	1.282848	0.016828
*CASP1*	18,518.75	33,218.6	0.843004	0.002609	39,462.42	1.091493	0.027117
*THBS4*	221.55	3.4	−6.02595	0.031067	<1	−7.79149	0.030685

*Average expression is presented as TPM. Positive log-2 ratios indicate upregulation in CTCL samples and negative log-2 ratios indicate downregulation in CTCL samples. p Values from Wald’s t-test are presented*.

**Table 2:** Genes with statistically significant differences in expression both between benign skin dermatoses vs. all ≥2009 CTCL samples (left panel) and between benign skin lesions vs. ≥2009 stage IV CTCL samples (right panel).

Genes	Average of benign [transcripts per million (TPM)]	Average of all CTCL (TPM)	log2 ratio of all CTCL vs. benign	*p* Value of all CTCL vs. benign	Average of stage IV CTCL (TPM)	log2 ratio of stage IV CTCL vs. benign	*p* Value of stage IV CTCL vs. benign
*GTSF1*	<1	1,755.165	10.77739	7.05E−05	2,350.786	11.19893	0.021712
*TOX*	410.12	2,315.593	2.497264	9.6E−10	3,506.295	3.095829	0.000662
*LTA*	189.1833	1,059.592	2.485652	1.6E−05	1,438.912	2.927121	0.016594
*CCR4*	1375.88	6,972.679	2.341358	4.11E−06	8,994.879	2.708749	0.002939
*FYB*	1337.875	6,662.626	2.316148	3.26E−13	10,651.85	2.993088	3.22E−05
*ITK*	140.1125	683.6844	2.286745	1.5E−05	765.1688	2.449192	0.005206
*SKAP1*	373.9	1,741.68	2.219756	1.47E−06	2,487.344	2.733882	0.000207
*LEF1*	161.0417	665.3026	2.046577	1.8E−05	769.1615	2.255853	0.000553
*MMP9*	149.2333	509.9797	1.77287	0.016193	1,277.873	3.098102	0.042027
*IL21R*	256.3111	805.0809	1.651238	0.000823	867.8737	1.759589	0.023029
*SH2D1A*	162.8714	504.3029	1.630557	0.000264	422.3357	1.374657	0.02604
*MDM4*	69.4	206.8441	1.575536	0.034407	269.8571	1.959188	0.037649
*ZAP70*	915.675	2616.744	1.514865	0.000397	2,726.044	1.573901	0.032018
*TRAF1*	221.0818	621.6043	1.491416	0.000464	862.61	1.964128	0.024488
*EED*	1110.654	3,032.809	1.449245	1.92E−07	3,058.529	1.461429	0.005258
*IL7R*	523.8217	1,253.655	1.258992	0.000674	1,282.056	1.291311	0.027339
*IRF4*	192.3	457.9822	1.251933	1.51E−05	468.6125	1.285037	0.00781
*PTPN6*	746.3091	1,754.427	1.233155	7.66E−06	1,674.979	1.166298	0.012107
*PILRB*	2562.259	5,979.205	1.222537	5.22E−07	5,874.39	1.197023	0.001808
*STAG3*	1118.487	2,561.473	1.195425	7.14E−05	3,466.835	1.632071	0.001586
*LCP2*	535.9733	1,203.391	1.166873	0.000358	1,290.937	1.268185	0.007005
*STAT1*	1714.134	3,738.915	1.125139	9.17E−05	4,591.761	1.421567	0.024235
*IL32*	2781.636	5,963.653	1.100262	0.000424	6,458.989	1.215375	0.005436
*CD52*	39313.92	82,527.71	1.069838	0.000555	87,585.14	1.155646	0.004942
*NFKB2*	320.1294	661.9056	1.04797	0.001318	719.4333	1.168206	0.011523
*STAT2*	324.7545	668.2443	1.041026	0.00217	1,074.281	1.72595	0.043189
*RAC2*	4323.963	8,859.232	1.034828	0.012875	12,229.12	1.499894	0.003941
*CCL5*	18304.19	37,008.56	1.015685	0.006721	43,941.66	1.263415	0.003185
*CNOT3*	268.192	538.7935	1.006466	0.000799	718.7158	1.422155	0.01338
*HDAC1*	1224.993	2441.145	0.994785	5.61E−05	3,158.984	1.366688	0.004638
*ZFX*	116.128	228.9473	0.979299	0.000605	259.8667	1.162056	0.048005
*MTF2*	217.3882	382.8481	0.816498	0.007382	369.1313	0.76386	0.032394
*ANKRD11*	987.6292	1,734.318	0.812327	0.000253	2,046.583	1.051176	0.033996
*SUZ12*	340.216	573.6457	0.753709	0.002437	703.375	1.047843	0.030192
*NUB1*	5078.115	8,463.066	0.736887	0.017489	9,530.422	0.908247	0.035693
*ZBTB16*	1100.665	1,818.246	0.724172	0.035544	2,833.213	1.364063	0.020919
*WWOX*	953.3938	1,551.22	0.70226	0.039474	1,921.327	1.010959	0.039726
*SERPINB13*	4,197.017	2,456.495	−0.77276	0.025735	2,005.013	−1.06575	0.010905
*SERPINB3*	12,386.85	5,653.013	−1.13172	0.012455	4,564.794	−1.44019	0.008324
*PSORS1C2*	56,092.96	15,415.36	−1.86345	0.001104	19,264.48	−1.54188	0.003512
*SERPINB4*	37,557.08	9,788.951	−1.93986	0.010726	8,392.617	−2.16189	0.01035
*EP400*	1,879.341	2,833.862	0.592543	4.28E−03	3,732.626	0.989964	0.021786
*TRRAP*	113.8296	154.2161	0.438078	1.14E−03	204.5421	0.845522	0.039173
*NFKB1*	498.076	620.1367	0.31622	2.43E−02	915.6556	0.878439	0.034389
*MAX*	8,108.728	8,575.909	0.080814	1.76E−03	11,353.27	0.48556	0.039695

*In light gray are presented four additional genes achieving statistical significance when using ≥2009 FFPE samples only (i.e., excluding freshly obtained samples from the analysis). Average expression is presented as TPM. Positive log-2 ratios indicate upregulation in CTCL samples and negative log-2 ratios indicate downregulation in CTCL samples. *p* Values from Wald’s *t*-test are presented*.

Based on these combined results, 42/75 DEGs were confirmed in both analyses, which highlights significant robustness of these tests. Of course, many of the initially identified DEGs did not achieve statistical significance since the number of samples analyzed in each of these subanalyses (i.e., ≤2008 and ≥2009) was significantly smaller than when all the data were analyzed as one set. Moreover, based on the original TruSeq data, subgroup analysis showed consistency in log-2 ratios between ≤2008 and ≥2009 CTCL samples. Indeed, rank–rank correlation when comparing benign dermatoses vs. all FFPE CTCL samples was ρ = 0.71 (strong; *p* < 10^−16^), while this indicator was ρ = 0.55 (medium; *p* < 10^−16^) when comparison was made between benign dermatoses and stage IV FFPE biopsies.

### Clustering Analysis of All FFPE CTCL Samples Using Subgroup Analyses Based on the Year of Biopsy and CTCL Clinical Cancer Stage

**Figure 5:**
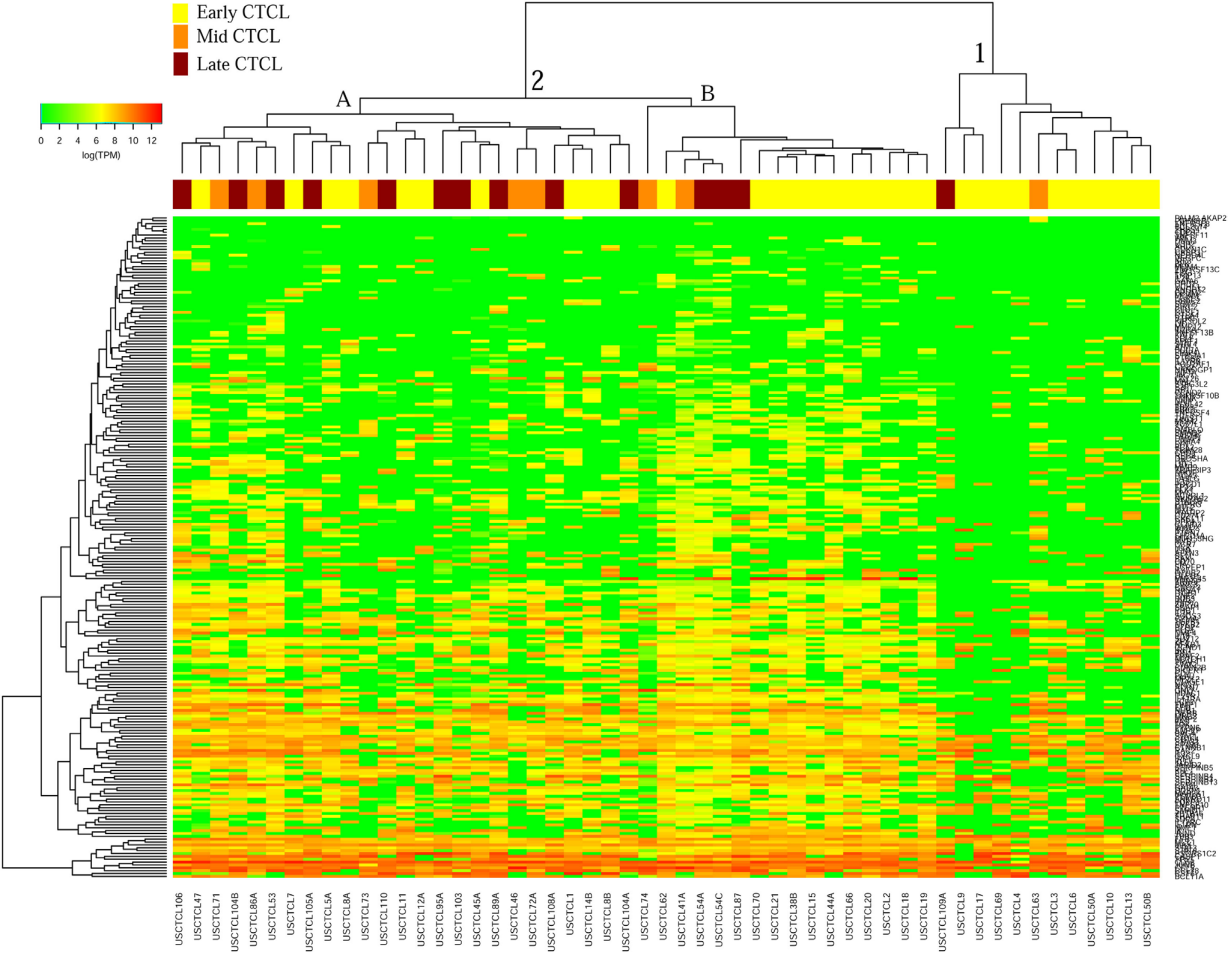
Unsupervised hierarchical clustering analysis based on TruSeq targeted RNA gene expression analysis of 284 select genes in ≤2008 early-stage (stage ≤IIA, yellow), mid-stage (stages IIB and III, orange), and late-stage (stage IV, dark red) formalin-fixed paraffin-embedded cutaneous T-cell lymphoma (CTCL) samples. A color key refers to gene expression in log(transcripts per million).

**Figure 6:**
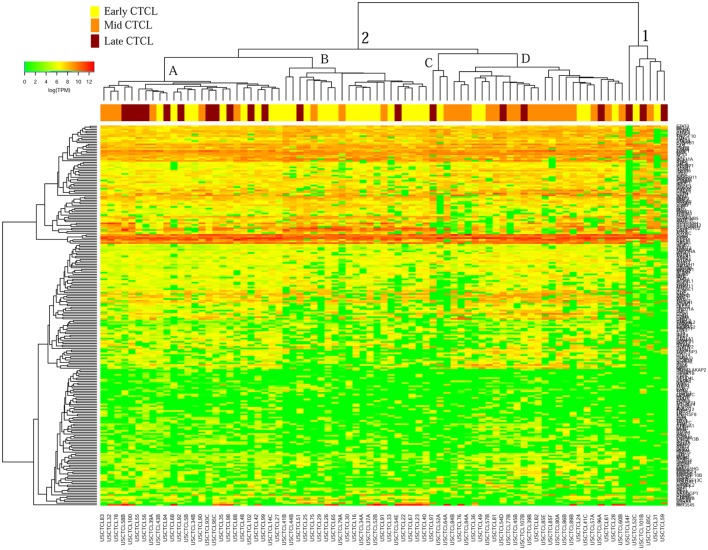
Unsupervised hierarchical clustering analysis based on TruSeq targeted RNA gene expression analysis of 284 select genes in ≥2009 early-stage (stage ≤IIA, yellow), mid-stage (stages IIB and III, orange), and late-stage (stage IV, dark red) formalin-fixed paraffin-embedded cutaneous T-cell lymphoma (CTCL) samples. A color key refers to gene expression in log(transcripts per million).

We then performed unsupervised hierarchical clustering analysis for early (≤IIA) vs. mid (IIB and III) vs. late (IV) stage CTCL for ≤2008 vs. ≥2009 samples. Similarly, we noted a significant molecular heterogeneity that was seen in our original report (41). However, for the ≤2008 CTCL FFPE samples (Figure 5), there were two major clusters. Cluster 1 had 12 samples, 10 of which were early-stage CTCL biopsies (10/31 of the total early-stage CTCL samples). Cluster 2 was rather heterogeneous with respect to its composition and could be subdivided into two subclusters: 2A, larger, with samples from all different stages and 2B with the well-defined subgroup of early-stage CTCL biopsies (10/11) on the right side of this subcluster. For ≥2009 samples (Figure 6), the distribution of samples was very heterogeneous as was seen in our earlier report (41).

### Identification of Differentially Expressed Genes (DEGs) in All FFPE CTCL Samples Using Subgroup Analyses Based on the Year of Biopsy and CTCL Clinical Cancer Stage

We next searched for the DEGs that were highlighted when we compared (1) early-stage (≤IIA) to mid and late CTCL stages (≥IIB) samples and (2) stage I vs. stage IV CTCL samples. Similarly, in this case, we analyzed ≤2008 and ≥2009 CTCL samples separately to test the robustness of the TruSeq results based on the year of skin biopsy. For ≤2008 samples, 12 genes were highlighted as being statistically significant: *TOX, EED*, and *LCP2* were upregulated in late-stage CTCL, while *ATXN7, CHD1, HUNK, TP63, KIT, JUNB, LTBP4, HDAC2*, and *OTUB2* were expressed preferentially in early-stage CTCL samples (Table 3). For ≥2009 samples, three different genes were identified: *SKAP1* and *GTSF1* were upregulated in late-stage CTCL, while *BCL11A* was upregulated in early-stage CTCL (Table 4). Overall, merging both subgroups, we validated three of the four genes observed in the full data set when performing the same analysis: *TOX* and *GTSF1* were upregulated in late-stage CTCL, and *LTBP4* was upregulated in early-stage CTCL.

**Table 3:** Genes with statistically significant differences in expression both between ≤2008 early-stage (≤IIA) vs. mid- and late-stage (≥IIB) formalin-fixed paraffin-embedded (FFPE) cutaneous T-cell lymphoma (CTCL) samples (left panel) and between ≤2008 stage I vs. stage IV FFPE CTCL samples (right panel).

Genes	Average of early CTCL [transcripts per million (TPM)]	Average of mid and late stages of CTCL (TPM)	log2 ratio early vs. mid and late stages of CTCL	*p* Value early vs. mid and late stages of CTCL	Average of stage I CTCL (TPM)	Average of stage IV CTCL (TPM)	log2 ratio of stage I vs. IV CTCL	*p* Value of stage I vs. IV CTCL
*LCP2*	1,279.782	2,740.947	1.098776	0.001332	1,351.527	2,396.473	0.826323	0.009062
*TOX*	2,286.086	4,579.155	1.002202	0.008709	2,343.758	4,502.264	0.941827	0.049764
*JUNB*	29,594.68	18,186.07	−0.7025	0.004493	28,825.75	19,214.87	−0.58513	0.040127
*EED*	5,408.253	2,911.241	−0.89353	0.025434	5,518.367	3,032.208	−0.86387	0.048117
*ATXN7*	936.3	494.4571	−0.92113	0.028724	932.8667	436.17	−1.09678	0.042606
*KIT*	1,493.838	629.5737	−1.24658	0.00016	1,529.729	695.6857	−1.13677	0.001263
*TP63*	13,320.81	5,028.95	−1.40535	0.005549	13,088.99	6,156.644	−1.08814	0.04105
*CHD1*	1,214.245	349.3444	−1.79734	0.017354	1,291.15	339.2167	−1.92838	0.017985
*HUNK*	512.48	112.225	−2.1911	0.046422	512.48	112.225	−2.1911	0.046422
*LTBP4*	732.3111	119.0667	−2.62069	0.014127	604.2125	142.45	−2.0846	0.029265
*HDAC2*	554.4625	83.8	−2.72607	0.025829	554.4625	111.4667	−2.31448	0.033172
*OTUB2*	8,321.478	1,190	−2.80588	0.012208	9,104.825	1,190	−2.93567	0.010448

*Average expression is presented as TPM. Positive log-2 ratios indicate upregulation in CTCL samples and negative log-2 ratios indicate downregulation in CTCL samples. p Values from Wald’s t-test are presented*.

**Table 4:** Genes with statistically significant differences in expression both between ≥2009 early stage (≤IIA) vs. mid and late stage (≥IIB) formalin-fixed paraffin-embedded (FFPE) cutaneous T-cell lymphoma (CTCL) samples (left panel) and between ≥2009 stage I vs. stage IV FFPE CTCL samples (right panel).

Genes	Average of early CTCL [transcripts per million (TPM)]	Average of mid and late stages of CTCL (TPM)	log2 ratio of early vs. mid and late stages of CTCL	*p* Value early vs. mid and late stages of CTCL	Average of stage I CTCL (TPM)	Average of stage IV CTCL (TPM)	log2 ratio of stage I vs. IV CTCL	*p* Value of stage I vs. IV CTCL
*GTSF1*	739.5667	3,103.076	2.068947	0.006142	434.14	2,350.786	2.436911	0.046242
*SKAP1*	1,361.83	2,508.23	0.881123	0.011364	1,350.189	2,487.344	0.881445	0.03311
*BCL11A*	12,154.45	7,382.605	−0.71928	0.011902	12,530.53	7660.058	−0.71002	0.037322

*Average expression is presented as TPM. Positive log-2 ratios indicate upregulation in CTCL samples and negative log-2 ratios indicate downregulation in CTCL samples. p values from Wald’s t-test are presented*.

This subgroup analysis showed moderate consistency in log-2 ratios ≤2008 and ≥2009 samples when comparing early vs. mid and late FFPE CTCL samples (ρ = 0.28; low; *p* < 10^−4^). However, there was no correlation when comparing stage I vs. stage IV FFPE tissues (ρ = 0.06; no correlation; *p* = 0.36).

### Comparison of the TruSeq Data Quality in FFPE vs. Freshly Obtained Snap-Frozen Samples

With respect to the RINs, a measure of sample quality prior to conducting the TruSeq analysis, we observed lower RINs for FFPE samples than freshly obtained snap-frozen samples. However, these RINs were within the expected range for FFPE samples (56, 57). RINs were much higher in fresh samples than in the FFPE samples, as expected (fresh: mean 6.1, 95% CI 5.5–6.8; FFPE: mean 2.4, 95% CI 2.3–2.5, *p* < 10^−6^ with MCMC). RNA libraries were also more concentrated in the freshly obtained samples (fresh: mean 227 ng/µL, 95% CI 110–239; FFPE: mean 64 ng/µL, 95% CI 53–75; *p* = 0.0012 with MCMC). There was no difference in RINs between ≤2008 and ≥2009 FFPE samples [≤2008: mean 2.3, 95% confidence interval (95% CI) 2.2–2.3; ≥2009: mean 2.4, 95% CI 2.3–2.6; *p* = 0.92 with MCMC]. RNA libraries were less concentrated in ≤2008 FFPE samples vs. ≥2009 samples, possibly explaining in part the lower TruSeq sequencing depth in ≤2008 samples (≤2008: mean 44 ng/µL, 95% CI 33–55; ≥2009: mean 76 ng/µL, 95% CI 60–92; *p* = 0.0007 with MCMC).

**Figure 7:**
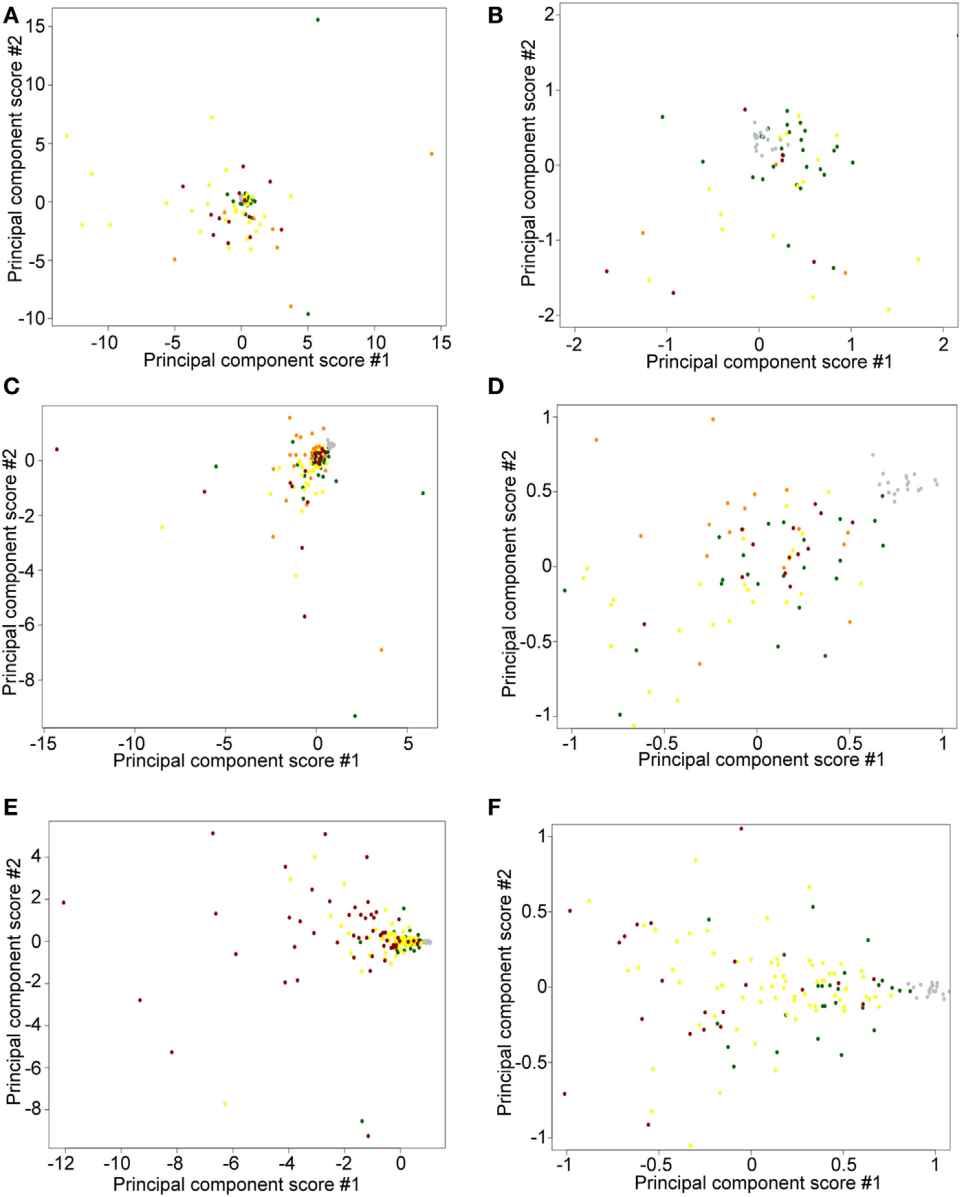
Principal component score plots. **(A,B)** First and second principal component scores of normal/benign (green), freshly obtained and liquid nitrogen snapped-frozen cutaneous T-cell lymphoma (CTCL) (gray), ≤2008 early-stage formalin-fixed paraffin-embedded (FFPE) CTCL (yellow), ≤2008 mid-stage FFPE CTCL (orange), and ≤2008 advanced stage FFPE CTCL (red) samples are plotted. **(C,D)** First and second principal component scores of normal/benign (green), freshly obtained and liquid nitrogen snapped-frozen CTCL (gray), ≥2009 early-stage FFPE CTCL (yellow), ≥2009 mid-stage FFPE CTCL (orange), and ≥2009 advanced stage FFPE CTCL (red) samples are plotted. **(E,F)** First and second principal component scores of normal/benign (green), freshly obtained, and liquid nitrogen snapped-frozen CTCL (gray), ≤2008 FFPE CTCL (red), and ≥2009 FFPE CTCL (yellow) samples are plotted.

To detect possible batch effects, we performed PCA on TPM data. We aimed to determine whether (1) freshly obtained flash snap-frozen samples cluster together and (2) ≤2008 and ≥2009 FFPE samples cluster in different areas. We observed a tight cluster of fresh CTCL samples (gray), whether using ≤2008 (Figures 7A,B) or ≥2009 (Figures 7C,D) FFPE CTCL samples, indicating that differences in preprocessing protocols might explain these findings (tight associations in clustering analyses). However, these freshly obtained samples were also in close spatial proximity to normal/benign samples and a number of FFPE samples. When comparing ≤2008 and ≥2009 FFPE samples, we observed no clear clusters (Figures 7E,F), but rather two loose associations. First, many newer samples (≥2009) were clustering around the center of the distribution, toward normal/benign and freshly obtained samples, indicating less preprocessing batch effect. Second, samples showing greater variability were mostly older samples (≤2008), indicating that there might be some processing batch effect among these. Taken together, these findings may explain why performing individual subgroup analyses enabled us to uncover additional DEGs.

## Discussion

In this study, we used subgroup analysis to determine whether older ≤2008 FFPE samples, which were sequenced at a lower depth on the TruSeq platform, were comparable to those obtained ≥2009. In this study, we also systematically analyzed sample processing biases based on the year of biopsy and the nature (i.e., FFPE vs. freshly obtained snap frozen) of the samples. Clustering analysis showed that ≤2008 and ≥2009 samples clustered equally well to the full data set and, furthermore, in a number of instances they demonstrated even better defined clusters. In particular, for ≤2008 samples, clusters were more reminiscent of the three clusters found in the landmark Boston CTCL cohort (3, 32, 48) when looking at all samples. There was also a better discrimination between benign and stage IV CTCL samples in ≤2008 samples than in the ≥2009 samples. Both analyses produced nearly identical trends and findings. Specifically, both analyses enriched nearly identical DEGs when comparing benign vs. (1) stage I–IV and (2) stage IV (alone) CTCL samples. Importantly, in this subgroup analysis, we recapitulated most of the targets seen within the full data set. Results obtained using either ≤2008 or ≥2009 samples were strongly correlated. Known upregulated targets in CTCL vs. benign dermatoses were validated, including *TOX, FYB, LEF*, and *STAT* signaling genes, inflammatory interleukins, *NF-κB* pathway signaling members, cancer testis genes, etc. We had previously reviewed in detail how these genes relate to the biology of CTCL tumorigenesis (3, 31).

Furthermore, this subgroup analysis enabled us to discover additional genes, which did not reach statistical significance in the full data set analysis. One may find it to be counterintuitive. However, indeed, despite the inherently decreased power, potential reasons why additional DEGs can be identified through subgroup analysis may include reduced variability on a per-sample basis due to increased in-group similarity and removal of outliers in some groups.

Those new DEGs included CTCL-upregulated *BCL11A* (regulation of RNA transcription), *SELL* (cell adhesion molecule in the selectin family), *IRF1* (Interferon transcription factor), *SMAD1* (*BMP* signaling and gene expression), *CASP1* (caspase involved in proteolysis), *BIRC5* (inhibitor of apoptosis, survivin), *MAX* (Myc-associated transcription factor), and CTCL-downregulated *MDM4* (negative regulator of p53), *SERPINB3* (serine protease involved in inflammatory response), and *THBS4* (cell-cell and cell-matrix interactions) genes. Of note, *THBS4* promoter was previously found to be frequently hypermethylated in 52% of CTCL samples, which leads to the downregulation in expression of this tumor suppressor gene (58). *CASP1* single-nucleotide polymorphisms were associated with changes in NF-κB signaling and development of other non-Hodgkin lymphomas, including diffuse-large B cell lymphomas and small lymphocytic lymphoma/chronic lymphocytic leukemia (59).

By using the full data set analysis, we found significant heterogeneity in our clusters (41). When we performed clustering on ≤2008 or ≥2009 FFPE CTCL samples, we still did not obtain three clusters that were previously described in the historic Boston cohort of CTCL patients (3, 32, 48). However, in this study of subgroup analyses, we noted less heterogeneity than we observed in the full data set analysis (41). PCA results also supported this conclusion. Indeed, in this subgroup analysis, samples of similar clinical disease stages were most often grouped together.

Subsequently, when we studied the DEGs enriched in both (1) early vs. mid and late CTCL and (2) stage I vs. stage IV disease, four genes were differentially expressed: *TOX* (involved in chromatin processes and T-cell development), *FYB* (T-cell adaptor protein), and *GTSF1* (germ cell maintenance) were upregulated, and *LTBP4* (latent TGF-beta binding protein) was downregulated in later CTCL stages. By merging subgroup analysis of ≤2008 and ≥2009 FFPE samples, our targets included *TOX, GTSF1*, and *LTBP4* as well. In particular, *TOX* overexpression is a hallmark of poor prognosis in CTCL, although low level of *TOX* expression has been previously reported in benign dermatoses (31, 60). *TOX* and *GTSF1* are aberrantly expressed developmental and meiotic genes that can prognosticate CTCL progression toward advanced disease (29, 31, 34). We also found that *EED* (Polycomb complex member expressed in embryonic stem cells), *SKAP1* (T-cell adhesion), and *LCP2* (T-cell receptor-mediated signaling) were upregulated in advanced CTCL stages. Surprisingly, we also found multiple genes with higher expression in early-stage tumors. These included *BCL11A* (see above), *ATXN7* (chromatin remodeling, *AKT* signaling), *HUNK* (*AMPK*-related kinase), *CHD1* (chromatin remodeling), *TP63* (transcription factor), *KIT* (receptor tyrosine kinase), *JUNB* (transcription factor), *HDAC2* (histone deacetylase), and *OTUB2* (deubiquitinase, inhibits proteolysis). Based on these combined results, transcription factors, chromatin remodelers, and global cell signaling processes are upregulated early in the disease, while in the advanced stages of CTCL, T-cell-specific genes, inflammatory mediators, and stem cell/germ cell maintenance genes appear to be driving cancer progression. These results further argue that subgroup analysis can often yield additional clues into the biology of cancers.

Formalin-fixed paraffin-embedded samples have RNA of lesser quality than the freshly obtained snap-frozen samples (61). However, FFPE samples are much easier to obtain in the clinical setting, have longer storage half-life, and are suitable for immunohistochemistry in a clinical pathology lab (62). Our FFPE RINs were comparable to those obtained in previous studies (56, 57). No matter if we performed subgroup analyses or full data set analysis, fresh samples tightly clustered together. While PCA revealed that fresh samples were spatially closer together, indicating some preprocessing batch effect, they remained in the proximity to other normal/benign and FFPE CTCL samples and were not clustering as outliers by themselves. However, this observed batch effect did not affect the determination of DEGs when analyzing all ≥2009 samples (fresh and FFPE biopsies) vs. ≥2009 FFPE samples alone. Other reports comparing freshly obtained frozen samples to FFPE samples showed a strong correlation (ρ > 0.70) in gene expression analysis (63). Formalin acts as a crosslinking agent for protein–protein, DNA–protein, and RNA–protein interactions (64). Crosslinking nucleic acid to proteins has its advantages in molecular medicine and is especially useful in characterizing transcription factor binding sites *via* chromatin immunoprecipitation (65) or RNA–protein interactions using RNA immunoprecipitation (66). In this study, we have successfully applied TruSeq targeted RNA sequencing to CTCL samples, both fresh and FFPE. A recent, direct comparison of TruSeq-analyzed RNA obtained from matched FFPE vs. fresh samples produced strongly correlated gene expression findings (*R*^2^ > 0.70) (67). Interestingly, previous studies showed that the RINs can range from 2.2 to 2.8 (median 2.3) for FFPE samples and 3.8 to 8.0 (median 6.8) for freshly obtained samples (67), which is consistent with our findings detailed in this report. In the study by Graw et al., illumina sequence reads between FFPE and freshly obtained matched samples showed a 0.33% error rate (67), which is consistent to previous reports for identical samples processed on the Illumina platform, when a 0.30% error rate was reported (68). In summary, our results indicate that performing targeted gene expression studies on the TruSeq platform from FFPE samples is a viable option that can be used in the real-life, clinical medicine setting.

## Ethics Statement

All patients were enrolled in the study in accordance with the IRB-approved protocols: PA12-0267, PA12-0497, and Lab97-256 at the MD Anderson Cancer Center (MDACC) and A09-M106-13A and 13-201-GEN at McGill University/McGill University Health Centre (MUHC). This study was carried out in accordance with the recommendations of the Research Ethics Board of the McGill University/McGill University Health Centre with written informed consent from all subjects in accordance with the Declaration of Helsinki. This study was carried out in accordance with the recommendations of the MD Anderson Cancer Center (MDACC) Research Ethics Board, which exempted us from obtaining written informed consent from patients, who earlier signed a hospital consent allowing their stored biopsy samples to be used for research.

## Author Contributions

PL, EN, MT, LM, AW, DS, XN, NP, MG, MD, and IL procured and analyzed patient sample data presented in this paper. PL, EN, and IL performed bioinformatic and statistical analyses. MT and AW performed pathological analysis of the original skin samples. PL, EN, MT, LM, AW, DS, XN, NP, MG, MD, and IL wrote the paper. NP, LM, MG, DS, MD, and IL supervised the study.

## Conflict of Interest Statement

The authors declare that the research was conducted in the absence of any commercial or financial relationships that could be construed as a potential conflict of interest. The reviewer YG and handling editor declared their shared affiliation.

## References

[1] Willemze R Jaffe ES Burg G Cerroni L Berti E Swerdlow SH WHO-EORTC classification for cutaneous lymphomas. Blood (2005) 105:3768–85. 10.1182/blood-2004-09-3502 15692063

[2] Han T Abdel-Motal UM Chang DK Sui J Muvaffak A Campbell J Human anti-CCR4 minibody gene transfer for the treatment of cutaneous T-cell lymphoma. PLoS One (2012) 7:e44455. 10.1371/journal.pone.0044455 22973452 PMC3433438

[3] Litvinov IV Netchiporouk E Cordeiro B Dore MA Moreau L Pehr K The use of transcriptional profiling to improve personalized diagnosis and management of cutaneous T-cell lymphoma (CTCL). Clin Cancer Res (2015) 21:2820–9. 10.1158/1078-0432.CCR-14-3322 25779945 PMC4470792

[4] Kirsch IR Watanabe R O’Malley JT Williamson DW Scott LL Elco CP TCR sequencing facilitates diagnosis and identifies mature T cells as the cell of origin in CTCL. Sci Transl Med (2015) 7:308ra158. 10.1126/scitranslmed.aaa9122 26446955 PMC4765389

[5] Ghazawi FM Netchiporouk E Rahme E Tsang M Moreau L Glassman S Comprehensive analysis of cutaneous T-cell lymphoma (CTCL) incidence and mortality in Canada reveals changing trends and geographic clustering for this malignancy. Cancer (2017) 123(18):3550–67. 10.1002/cncr.30758 28493286

[6] Litvinov IV Tetzlaff MT Rahme E Habel Y Risser DR Gangar P Identification of geographic clustering and regions spared by cutaneous T-cell lymphoma in Texas using 2 distinct cancer registries. Cancer (2015) 121:1993–2003. 10.1002/cncr.29301 25728286 PMC4457714

[7] Litvinov IV Tetzlaff MT Rahme E Jennings MA Risser DR Gangar P Demographic patterns of cutaneous T-cell lymphoma incidence in Texas based on two different cancer registries. Cancer Med (2015) 4:1440–7. 10.1002/cam4.472 26136403 PMC4567029

[8] Moreau JF Buchanich JM Geskin JZ Akilov OE Geskin LJ. Non-random geographic distribution of patients with cutaneous T-cell lymphoma in the Greater Pittsburgh Area. Dermatol Online J (2014) 20(7):13030. 25046454

[9] Barba G Matteucci C Girolomoni G Brandimarte L Varasano E Martelli MF Comparative genomic hybridization identifies 17q11.2 approximately q12 duplication as an early event in cutaneous T-cell lymphomas. Cancer Genet Cytogenet (2008) 184:48–51. 10.1016/j.cancergencyto.2008.03.007 18558289

[10] Caprini E Cristofoletti C Arcelli D Fadda P Citterich MH Sampogna F Identification of key regions and genes important in the pathogenesis of sezary syndrome by combining genomic and expression microarrays. Cancer Res (2009) 69:8438–46. 10.1158/0008-5472.CAN-09-2367 19843862

[11] Fischer TC Gellrich S Muche JM Sherev T Audring H Neitzel H Genomic aberrations and survival in cutaneous T cell lymphomas. J Invest Dermatol (2004) 122:579–86. 10.1111/j.0022-202X.2004.22301.x 15086538

[12] Karenko L Sarna S Kahkonen M Ranki A. Chromosomal abnormalities in relation to clinical disease in patients with cutaneous T-cell lymphoma: a 5-year follow-up study. Br J Dermatol (2003) 148:55–64. 10.1046/j.1365-2133.2003.05116.x 12534595

[13] Laharanne E Oumouhou N Bonnet F Carlotti M Gentil C Chevret E Genome-wide analysis of cutaneous T-cell lymphomas identifies three clinically relevant classes. J Invest Dermatol (2010) 130:1707–18. 10.1038/jid.2010.8 20130593

[14] Mao X Lillington D Scarisbrick JJ Mitchell T Czepulkowski B Russell-Jones R Molecular cytogenetic analysis of cutaneous T-cell lymphomas: identification of common genetic alterations in Sezary syndrome and mycosis fungoides. Br J Dermatol (2002) 147:464–75. 10.1046/j.1365-2133.2002.04966.x 12207585

[15] Mao X Lillington DM Czepulkowski B Russell-Jones R Young BD Whittaker S Molecular cytogenetic characterization of Sezary syndrome. Genes Chromosomes Cancer (2003) 36:250–60. 10.1002/gcc.10152 12557225

[16] Mao X McElwaine S Functional copy number changes in Sezary syndrome: toward an integrated molecular cytogenetic map III. Cancer Genet Cytogenet (2008) 185:86–94. 10.1016/j.cancergencyto.2008.05.006 18722877

[17] Prochazkova M Chevret E Mainhaguiet G Sobotka J Vergier B Belaud-Rotureau MA Common chromosomal abnormalities in mycosis fungoides transformation. Genes Chromosomes Cancer (2007) 46:828–38. 10.1002/gcc.20469 17584911

[18] Salgado R Servitje O Gallardo F Vermeer MH Ortiz-Romero PL Karpova MB Oligonucleotide array-CGH identifies genomic subgroups and prognostic markers for tumor stage mycosis fungoides. J Invest Dermatol (2010) 130:1126–35. 10.1038/jid.2009.306 19759554

[19] Shapiro PE Warburton D Berger CL Edelson RL. Clonal chromosomal abnormalities in cutaneous T-cell lymphoma. Cancer Genet Cytogenet (1987) 28:267–76. 10.1016/0165-4608(87)90213-5 3497708

[20] Thangavelu M Finn WG Yelavarthi KK Roenigk HH Jr Samuelson E Peterson L Recurring structural chromosome abnormalities in peripheral blood lymphocytes of patients with mycosis fungoides/Sezary syndrome. Blood (1997) 89:3371–7. 9129044

[21] van Doorn R van Kester MS Dijkman R Vermeer MH Mulder AA Szuhai K Oncogenomic analysis of mycosis fungoides reveals major differences with Sezary syndrome. Blood (2009) 113:127–36. 10.1182/blood-2008-04-153031 18832135

[22] Vermeer MH van Doorn R Dijkman R Mao X Whittaker S van Voorst Vader PC Novel and highly recurrent chromosomal alterations in Sezary syndrome. Cancer Res (2008) 68:2689–98. 10.1158/0008-5472.CAN-07-6398 18413736

[23] Wain EM Mitchell TJ Russell-Jones R Whittaker SJ. Fine mapping of chromosome 10q deletions in mycosis fungoides and sezary syndrome: identification of two discrete regions of deletion at 10q23.33-24.1 and 10q24.33-25.1. Genes Chromosomes Cancer (2005) 42:184–92. 10.1002/gcc.20115 15540164

[24] Wang L Ni X Covington KR Yang BY Shiu J Zhang X Genomic profiling of Sezary syndrome identifies alterations of key T cell signaling and differentiation genes. Nat Genet (2015) 47:1426–34. 10.1038/ng.3444 26551670 PMC4829974

[25] Ungewickell A Bhaduri A Rios E Reuter J Lee CS Mah A Genomic analysis of mycosis fungoides and Sezary syndrome identifies recurrent alterations in TNFR2. Nat Genet (2015) 47:1056–60. 10.1038/ng.3370 26258847 PMC6091217

[26] Sandoval J Diaz-Lagares A Salgado R Servitje O Climent F Ortiz-Romero PL MicroRNA expression profiling and DNA methylation signature for deregulated microRNA in cutaneous T-cell lymphoma. J Invest Dermatol (2015) 135:1128–37. 10.1038/jid.2014.487 25405321

[27] McGirt LY Jia P Baerenwald DA Duszynski RJ Dahlman KB Zic JA Whole-genome sequencing reveals oncogenic mutations in mycosis fungoides. Blood (2015) 126:508–19. 10.1182/blood-2014-11-611194 26082451 PMC4513251

[28] da Silva Almeida AC Abate F Khiabanian H Martinez-Escala E Guitart J Tensen CP The mutational landscape of cutaneous T cell lymphoma and Sezary syndrome. Nat Genet (2015) 47:1465–70. 10.1038/ng.3442 26551667 PMC4878831

[29] Huang Y Litvinov IV Wang Y Su MW Tu P Jiang X Thymocyte selection-associated high mobility group box gene (TOX) is aberrantly over-expressed in mycosis fungoides and correlates with poor prognosis. Oncotarget (2014) 5:4418–25. 10.18632/oncotarget.2031 24947046 PMC4147334

[30] Litvinov IV Cordeiro B Fredholm S Odum N Zargham H Huang Y Analysis of STAT4 expression in cutaneous T-cell lymphoma (CTCL) patients and patient-derived cell lines. Cell Cycle (2014) 13:2975–82. 10.4161/15384101.2014.947759 25486484 PMC4614388

[31] Litvinov IV Cordeiro B Huang Y Zargham H Pehr K Dore MA Ectopic expression of cancer testis antigens in cutaneous T-cell lymphoma (CTCL) patients. Clin Cancer Res (2014) 20(14):3799–808. 10.1158/1078-0432.CCR-14-0307 24850846 PMC4863442

[32] Litvinov IV Jones DA Sasseville D Kupper TS. Transcriptional profiles predict disease outcome in patients with cutaneous T-cell lymphoma. Clin Cancer Res (2010) 16:2106–14. 10.1158/1078-0432.CCR-09-2879 20233883 PMC2853253

[33] Litvinov IV Kupper TS Sasseville D. The role of AHI1 and CDKN1C in cutaneous T-cell lymphoma progression. Exp Dermatol (2012) 21:964–6. 10.1111/exd.12039 23171462 PMC5215484

[34] Litvinov IV Netchiporouk E Cordeiro B Zargham H Pehr K Gilbert M Ectopic expression of embryonic stem cell and other developmental genes in cutaneous T-cell lymphoma. Oncoimmunology (2014) 3:e970025. 10.4161/21624011.2014.970025 25941598 PMC4368148

[35] Litvinov IV Pehr K Sasseville D Connecting the dots in cutaneous T cell lymphoma (CTCL): STAT5 regulates malignant T cell proliferation via miR-155. Cell Cycle (2013) 12:2172–3. 10.4161/cc.25550 23803726 PMC3755065

[36] Litvinov IV Zhou Y Kupper TS Sasseville D Loss of BCL7A expression correlates with poor disease prognosis in patients with early-stage cutaneous T-cell lymphoma. Leuk Lymphoma (2013) 54(3):653–4. 10.3109/10428194.2012.717695 22856870

[37] Kopp KL Ralfkiaer U Nielsen BS Gniadecki R Woetmann A Odum N Expression of miR-155 and miR-126 in situ in cutaneous T-cell lymphoma. APMIS (2013) 121:1020–4. 10.1111/apm.12162 24033365

[38] Marstrand T Ahler CB Ralfkiaer U Clemmensen A Kopp KL Sibbesen NA Validation of a diagnostic microRNA classifier in cutaneous T-cell lymphomas. Leuk Lymphoma (2014) 55:957–8. 10.3109/10428194.2013.815352 23772646

[39] Ralfkiaer U Hagedorn PH Bangsgaard N Lovendorf MB Ahler CB Svensson L Diagnostic microRNA profiling in cutaneous T-cell lymphoma (CTCL). Blood (2011) 118:5891–900. 10.1182/blood-2011-06-358382 21865341 PMC3342856

[40] Ralfkiaer U Lindahl LM Litman T Gjerdrum LM Ahler CB Gniadecki R MicroRNA expression in early mycosis fungoides is distinctly different from atopic dermatitis and advanced cutaneous T-cell lymphoma. Anticancer Res (2014) 34(12):7207–17. 25503151

[41] Litvinov IV Tetzlaff MT Thibault P Gangar P Moreau L Watters AK Gene expression analysis in Cutaneous T-Cell Lymphomas (CTCL) highlights disease heterogeneity and potential diagnostic and prognostic indicators. Oncoimmunology (2017) 6:e1306618. 10.1080/2162402X.2017.1306618 28638728 PMC5468001

[42] Clark RA. Resident memory T cells in human health and disease. Sci Transl Med (2015) 7:269rv1. 10.1126/scitranslmed.3010641 25568072 PMC4425129

[43] Scarisbrick JJ Prince HM Vermeer MH Quaglino P Horwitz S Porcu P Effect of specific prognostic markers on survival and development of a prognostic model. J Clin Oncol (2015) 33:3766–73. 10.1200/JCO.2015.61.7142 26438120 PMC4979132

[44] Alberti-Violetti S Talpur R Schlichte M Sui D Duvic M Advanced-stage mycosis fungoides and Sezary syndrome: survival and response to treatment. Clin Lymphoma Myeloma Leuk (2015) 15:e105–12. 10.1016/j.clml.2015.02.027 25817937

[45] Talpur R Singh L Daulat S Liu P Seyfer S Trynosky T Long-term outcomes of 1,263 patients with mycosis fungoides and Sezary syndrome from 1982 to 2009. Clin Cancer Res (2012) 18:5051–60. 10.1158/1078-0432.CCR-12-0604 22850569 PMC3857608

[46] McGirt LY Baerenwald DA Vonderheid EC Eischen CM Early changes in miRNA expression are predictive of response to extracorporeal photopheresis in cutaneous T-cell lymphoma. J Eur Acad Dermatol Venereol (2015) 29:2269–71. 10.1111/jdv.12571 24909834 PMC4831701

[47] Kamstrup MR Gjerdrum LM Biskup E Lauenborg BT Ralfkiaer E Woetmann A Notch1 as a potential therapeutic target in cutaneous T-cell lymphoma. Blood (2010) 116:2504–12. 10.1182/blood-2009-12-260216 20538790

[48] Shin J Monti S Aires DJ Duvic M Golub T Jones DA Lesional gene expression profiling in cutaneous T-cell lymphoma reveals natural clusters associated with disease outcome. Blood (2007) 110:3015–27. 10.1182/blood-2006-12-061507 17638852 PMC2018675

[49] Danish HH Liu S Jhaveri J Flowers CR Lechowicz MJ Esiashvili N Validation of cutaneous lymphoma international prognostic index (CLIPI) for mycosis fungoides and Sezary syndrome. Leuk Lymphoma (2016) 57(12):2813–9. 10.3109/10428194.2016.1173210 27104864

[50] Leek JT Storey JD. Capturing heterogeneity in gene expression studies by surrogate variable analysis. PLoS Genet (2007) 3:1724–35. 10.1371/journal.pgen.0030161 17907809 PMC1994707

[51] Johnson WE Li C Rabinovic A. Adjusting batch effects in microarray expression data using empirical Bayes methods. Biostatistics (2007) 8:118–27. 10.1093/biostatistics/kxj037 16632515

[52] Price AL Patterson NJ Plenge RM Weinblatt ME Shadick NA Reich D. Principal components analysis corrects for stratification in genome-wide association studies. Nat Genet (2006) 38:904–9. 10.1038/ng1847 16862161

[53] Gower JC A general coefficient of similarity and some of its properties. Biometrics (1971) 27:857–74. 10.2307/2528823

[54] Murtagh F Legendre P Ward’s hierarchical agglomerative clustering method: which algorithms implement ward’s criterion? J Classif (2014) 31:274–95. 10.1007/s00357-014-9161-z

[55] Stacklies W Redestig H Scholz M Walther D Selbig J pcaMethods – a bioconductor package providing PCA methods for incomplete data. Bioinformatics (2007) 23:1164–7. 10.1093/bioinformatics/btm069 17344241

[56] von Ahlfen S Missel A Bendrat K Schlumpberger M. Determinants of RNA quality from FFPE samples. PLoS One (2007) 2:e1261. 10.1371/journal.pone.0001261 18060057 PMC2092395

[57] Ribeiro-Silva A Zhang H Jeffrey SS. RNA extraction from ten year old formalin-fixed paraffin-embedded breast cancer samples: a comparison of column purification and magnetic bead-based technologies. BMC Mol Biol (2007) 8:118. 10.1186/1471-2199-8-118 18154675 PMC2233637

[58] van Doorn R Zoutman WH Dijkman R de Menezes RX Commandeur S Mulder AA Epigenetic profiling of cutaneous T-cell lymphoma: promoter hypermethylation of multiple tumor suppressor genes including BCL7a, PTPRG, and p73. J Clin Oncol (2005) 23:3886–96. 10.1200/JCO.2005.11.353 15897551

[59] Langmead B Trapnell C Pop M Salzberg SL. Ultrafast and memory-efficient alignment of short DNA sequences to the human genome. Genome Biol (2009) 10:R25. 10.1186/gb-2009-10-3-r25 19261174 PMC2690996

[60] Huang Y Su MW Jiang X Zhou Y. Evidence of an oncogenic role of aberrant TOX activation in cutaneous T-cell lymphoma. Blood (2015) 125:1435–43. 10.1182/blood-2014-05-571778 25548321

[61] Scicchitano MS Dalmas DA Bertiaux MA Anderson SM Turner LR Thomas RA Preliminary comparison of quantity, quality, and microarray performance of RNA extracted from formalin-fixed, paraffin-embedded, and unfixed frozen tissue samples. J Histochem Cytochem (2006) 54:1229–37. 10.1369/jhc.6A6999.2006 16864893

[62] Perlmutter MA Best CJ Gillespie JW Gathright Y Gonzalez S Velasco A Comparison of snap freezing versus ethanol fixation for gene expression profiling of tissue specimens. J Mol Diagn (2004) 6:371–7. 10.1016/S1525-1578(10)60534-X 15507677 PMC1867483

[63] Mittempergher L de Ronde JJ Nieuwland M Kerkhoven RM Simon I Rutgers EJ Gene expression profiles from formalin fixed paraffin embedded breast cancer tissue are largely comparable to fresh frozen matched tissue. PLoS One (2011) 6:e17163. 10.1371/journal.pone.0017163 21347257 PMC3037966

[64] Werner M Chott A Fabiano A Battifora H. Effect of formalin tissue fixation and processing on immunohistochemistry. Am J Surg Pathol (2000) 24:1016–9. 10.1097/00000478-200007000-00014 10895825

[65] Solomon MJ Varshavsky A. Formaldehyde-mediated DNA-protein crosslinking: a probe for in vivo chromatin structures. Proc Natl Acad Sci U S A (1985) 82:6470–4. 10.1073/pnas.82.19.6470 2995966 PMC390738

[66] Au PC Helliwell C Wang MB. Characterizing RNA-protein interaction using cross-linking and metabolite supplemented nuclear RNA-immunoprecipitation. Mol Biol Rep (2014) 41:2971–7. 10.1007/s11033-014-3154-1 24493449

[67] Graw S Meier R Minn K Bloomer C Godwin AK Fridley B Robust gene expression and mutation analyses of RNA-sequencing of formalin-fixed diagnostic tumor samples. Sci Rep (2015) 5:12335. 10.1038/srep12335 26202458 PMC4511951

[68] Lefrancois P Euskirchen GM Auerbach RK Rozowsky J Gibson T Yellman CM Efficient yeast ChIP-Seq using multiplex short-read DNA sequencing. BMC Genomics (2009) 10:37. 10.1186/1471-2164-10-37 19159457 PMC2656530

